# Left atrial posterior wall isolation in addition to pulmonary vein isolation using a pentaspline catheter in pulsed-field ablation for atrial fibrillation: A systematic review and meta-analysis

**DOI:** 10.1016/j.hroo.2024.08.006

**Published:** 2024-09-28

**Authors:** Raymond Pranata, William Kamarullah, Giky Karwiky, Chaerul Achmad, Mohammad Iqbal

**Affiliations:** Department of Cardiology and Vascular Medicine, Faculty of Medicine, Universitas Padjadjaran, Hasan Sadikin General Hospital, Kota Bandung, Jawa Barat, Indonesia

**Keywords:** Ablation, Pulsed-field ablation, Atrial fibrillation, Left atrial posterior wall isolation, Pulmonary vein isolation, Additional ablation, Atrial tachycardia, Atrial arrhythmia, Catheter ablation

## Abstract

**Background:**

Persistent atrial fibrillation (AF) may require extensive ablation strategies. Left atrial posterior wall isolation (LAPWI) might address potential substrates for recurrence during pulsed-field ablation (PFA).

**Objective:**

This meta-analysis aimed to investigate the feasibility and effectiveness of LAPWI in addition to pulmonary vein isolation (PVI) using a pentaspline catheter in PFA for AF.

**Methods:**

Comprehensive search was conducted using PubMed, SCOPUS, ScienceDirect, and EuropePMC for studies reporting LAPWI+PVI using a pentaspline catheter in PFA ablation for AF. The primary outcome was atrial tachyarrhythmia (ATa) recurrence, defined as AF/atrial flutter/atrial tachycardia after blanking period.

**Results:**

There were 882 patients from 7 studies. The success rate of LAPWI was 100% using mean/median of 16 to 20 added PFA applications with no reported acute left atrial posterior wall reconnection and esophageal complications. In mean follow-up of 240 ± 91 days, ATa recurrence was 21% (95% CI 13%–29%; I^2^ = 84.8%) in the LAPWI+PVI group. Meta-regression analysis showed that age, left ventricular ejection fraction, and repeat procedure did not significantly influence ATa recurrence (*P* > .05). Each 1-mm increase in left atrial diameter, increases the chance of ATa recurrence by 6% (R^2^ = 100%, *P* < .001, I^2^ = 0%). Meta-analysis showed no difference in terms of ATa recurrence among LAPWI+PVI patients compared with those without LAPWI (odds ratio 0.78, 95% confidence interval 0.50–1.21, *P* = .27; I^2^ = 0%, *P* = .86). Procedure time and fluoroscopy time did not significantly differ (*P* > .05).

**Conclusion:**

LAPWI using a pentaspline catheter during PFA was feasible and did not prolong the procedure/fluoroscopy but did not reduce ATa recurrence. LAPWI may be considered during PFA, although the benefit is uncertain.

**Key Findings**

▪ LAPWI using a pentaspline catheter exclusively without additional point-by-point touch-up ablation during PFA was feasible with a 100% success rate.
▪ The addition of LAPWI to PVI during PFA did not reduce ATa recurrence.
▪ The recurrence of AF in LAPWI+PVI was significantly affected by LA diameter at baseline.
▪ Performing LAPWI on top of PVI does not significantly prolong the procedure or fluoroscopy time.
▪ LAPWI may be considered during PFA ablation without significantly prolonging the procedure, although the benefit is uncertain.

## Introduction

Pulmonary vein isolation (PVI) has been denoted as an essential part of catheter ablation for both paroxysmal and persistent atrial fibrillation (AF).^1^^,^^2^ Complete electrical isolation of the pulmonary veins (PVs) through lesions around the antra is the primary ablation objective in both paroxysmal and persistent AF. It is increasingly acknowledged that persistent AF commencement as well as maintenance may be reliant on mechanisms other than PV. As a result, more extensive ablation has been frequently considered in patients with persistent AF because of possible substrate beyond PVI, albeit with disappointing results.^3^^,^^4^ However, the long-term effectiveness of PVI in individuals with persistent AF is limited, with studies showing a success rate of 40% to 50%.^4^^,^^5^ The advent of pulsed-field ablation (PFA) enables single-shot ablation in patients with AF, creating a durable lesion in a short period of time; however, the recurrence of arrhythmia also remains high in patients with persistent AF.^6^ Thus, additional ablation techniques, especially those that can be performed without requiring a radiofrequency catheter for lesion formation, are of interest.

A study reporting findings from repeat ablation using high-density mapping after PVI with PFA showed that the incidence of atrial tachycardia was unexpectedly high compared with ablation using other modalities, and the substrate seemed to be associated with postablation macro–re-entry circuits.^7^ The mechanisms of atrial tachycardia were mostly re-entry, and the critical isthmus was located at the posterior wall in 62% of the patients, suggesting the potential role of prophylactic left atrial posterior wall isolation (LAPWI) in patients undergoing PVI with PFA. The posterior wall of the left atrium (LA) lies close to esophagus; thus, LAPWI using thermal energy increases the risk of esophageal complications. Unlike thermal ablation, PFA has myocardial tissue selectivity, thus sparing nerves, vasculature, and esophageal tissue, which may avoid esophageal complications during LAPWI.^8^, ^9^, ^10^, ^11^, ^12^, ^13^, ^14^ LAPWI also involves extensive lesion formation, and PFA was shown to prevent restrictive physiology, as the LA function indices were shown to recover with PFA but not with thermal ablation.^9^^,^^15^ The PersAFOne (Pulsed Fields for Persistent Atrial Fibrillation) study showed that LAPWI can be performed using a pentaspline PFA catheter alone, without the need for point-by-point ablation.^16^ This meta-analysis aimed to investigate the feasibility and effectiveness of LAPWI in addition to PVI using a pentaspline catheter exclusively in PFA for AF.

## Methods

### Protocol and registration

This systematic review was conducted in accordance with the Cochrane Handbook for Systematic Reviews of Interventions and reported based on the PRISMA (Preferred Reporting Items for Systematic Reviews and Meta-Analysis) statement. The protocol was registered at the PROSPERO, under identification number (CRD42024550514).

### Literature search strategy

**Figure 1:**
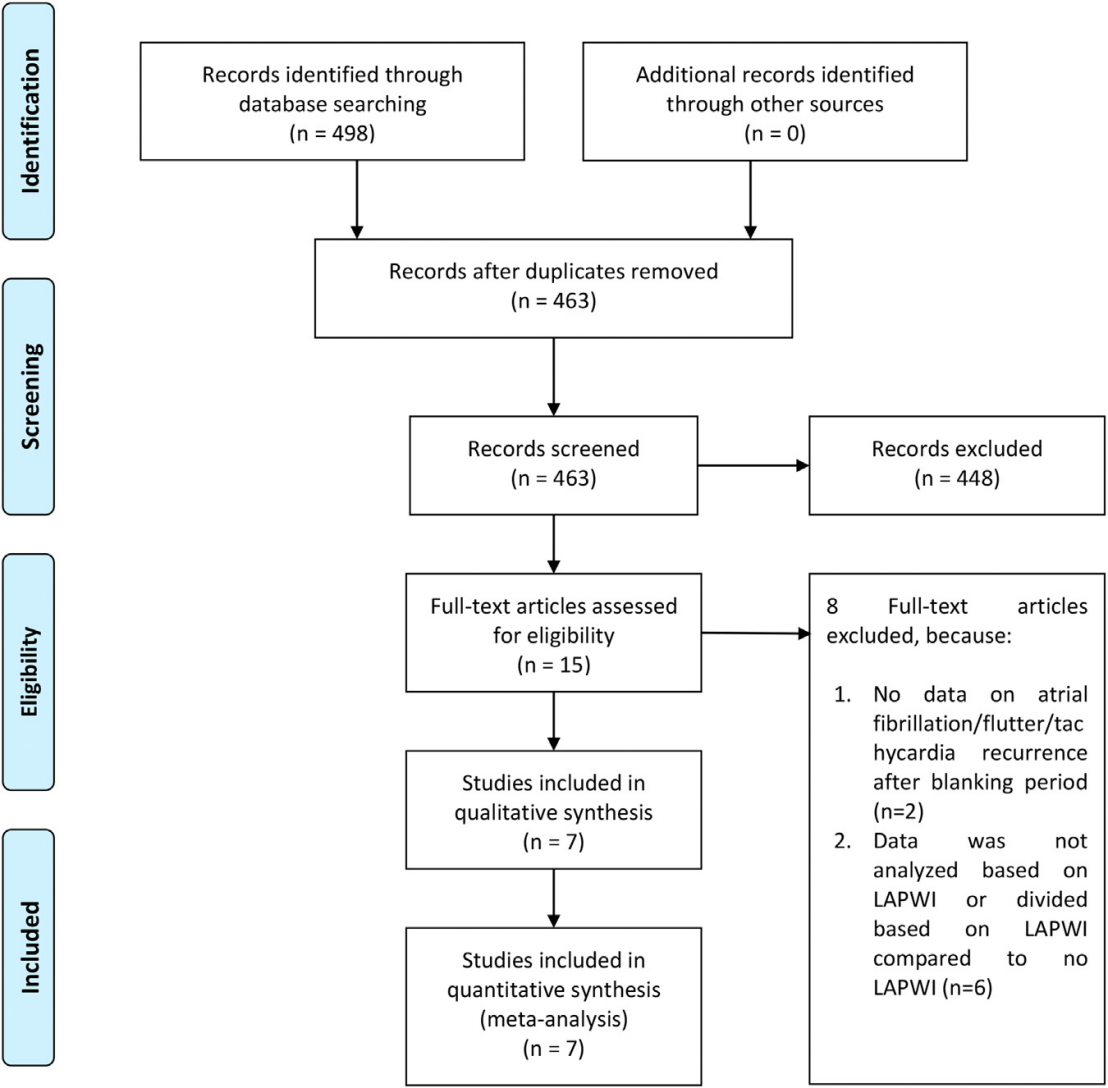
PRISMA (Preferred Reporting Items for Systematic Reviews and Meta-Analysis) flowchart. LAPWI = left atrial posterior wall isolation.

Two independent investigators conducted a systematic search of PubMed, SCOPUS, Europe PMC, and ScienceDirect from inception to May 2, 2024. The search query includes keywords and search phrases that involve (pulsed field ablation) AND ((left atrial posterior wall isolation) or (LAPWI) or (posterior wall ablation)) AND (atrial fibrillation). We employed the least number of keywords possible to optimize the initial area of inquiry and ensure that the greatest number of articles were recorded. To widen our search results, we also performed hand searches through the references of the included articles. The PRISMA criteria were implemented in our search, and Figure 1 depicts the search and screening processes.

### Study selection

In this meta-analysis, we incorporated studies reporting the success rates, atrial tachyarrhythmia (ATa) recurrences, procedure duration, and fluoroscopy duration of LAPWI+PVI PFA (single arm) and studies that compared LAPWI+PVI with those without LAPWI in terms of the aforementioned data. We also excluded case reports/series, editorials, comments, letters to the editor, animal studies, review papers, meta-analyses, and conference abstracts from our meta-analysis.

### Intervention vs control groups

The population was AF patients undergoing PVI using the PFA technique, which causes lesions in cardiac tissue nonthermally and in milliseconds via the irreversible electroporation process. The PFA included studies that utilized the pentaspline ablation catheter to conduct the ablation process. Exploratory PFA was carried out along the posterior wall in an overlapping manner between the superior aspect of the superior PV and the most inferior edges of the inferior PV using a pentaspline catheter in flower configuration. In non–single-arm studies, the intervention group referred to the group receiving LAPWI, which was performed using an electroanatomic mapping device or fluoroscopy guidance. The control group referred to group that did not receive LAPWI. The lack of electrograms collected on the pentaspline PFA catheter verified LAPWI. Our research protocol permit investigations encompassing additional ablation beyond PVI and LAPWI.

### Outcomes of interest

The primary outcome was ATa recurrence, defined as AF/atrial flutter/atrial tachycardia lasting longer than 30 seconds after ablation for at least 3 months (blanking period). Secondary outcomes were success rate, total procedure time (minutes), fluoroscopy time (minutes), and esophageal complications.

### Data extraction and risk-of-bias assessment

Data abstraction was carried out independently by 2 authors (R.P. and W.K.) using a form that detailed the baseline characteristics of the included studies (age, study design, study population), redo ablation at baseline, persistent AF percentage, success rate, average application of LAPWI, LAPWI reconnection, complications, procedural characteristics, LA size, left ventricular ejection fraction, and follow-up time.

We used the Newcastle-Ottawa Scale to independently assess the probability of bias for comparative observational studies. A study with a total score of 7 or above was considered bias-free. Research with a total score of 6 or lower was judged to be biased. Author discussions were used to resolve quality rating conflicts.

### Statistical analysis

In this meta-analysis, we used STATA 17 (StataCorp) to determine the overall effect size. The Mantel-Haenszel technique and the general inverse variance approach were used for dichotomous and continuous data, respectively. The odds ratio was employed to quantify binary comparison, while the mean difference was used to estimate continuous variable comparison as an effect unit. I^2^ was used to quantify the pooled estimate's heterogeneity. A value of >50% or a *P* value <.10 indicated statistically significant heterogeneity. The pooled effect size was calculated using the random-effects model, irrespective of heterogeneity. All statistical analyses were 2-sided, and a *P* value <.05 indicated statistical significance. Meta-regression analysis was performed using restricted maximum-likelihood method on the AF recurrence in LAPWI+PVI PFA group. Funnel-plot and Egger’s test for publication bias and small-study effect were not performed because there were only 3 comparative studies.

## Results

There were 882 patients from 7 studies.^17^, ^18^, ^19^, ^20^, ^21^, ^22^, ^23^ The baseline characteristics of the included studies were outlined in Tables 1 and 2. Most patients have persistent AF. The mean follow-up duration was 240 ± 91 days. There were no reported esophageal complications or atrioesophageal fistulas in the studies. Comparative studies including Gunawardene and colleagues,^17^ Schiavone and colleagues,^19^ and Turagam and colleagues^21^ have a low risk of bias (Newcastle-Ottawa Scale >7).

**Table 1:** Baseline characteristics of the included studies

Study	Sample size	Design	Inclusion criteria	Redo ablation at baseline (%)	Persistent AF (%)	Age (y)	LA diameter (mm)	LVEF (%)	Mean FU (d)
Badertscher 2024^22^	100	RO (SA)	Paroxysmal/persistent AF undergoing PVI+PWI	64	55	69	43	55	144
Davong 2023^23^	45	PO (SA)	De novo persistent AF undergoing LAPWI+PVI+mitral isthmus block	0	100	67	NA (LA volume 145 mL)	57	108
Gunawardene 2023^17^	79	PO	De novo and repeat ablation for LAPWI+PVI+additional ablation vs de novo for PVI only	73 vs 0	100	64 vs 69	45 vs 44	NA	354
Kueffer 2024^18^	215	PO (SA)	Paroxysmal/persistent AF or LA tachycardia undergoing LAPWI+PVI+additional substrate ablation	67.4	70.2	69.5	46.5	55	222
Schiavone 2024^19^	249	PO	Persistent AF undergoing LAPWI+PVI vs PVI only	17 vs 12	100	63.4	NA (LA volume 42.6 mL)	54	273
Sohns 2023^20^	10	PO (SA)	Paroxysmal/persistent AF undergoing LAPWI+PVI	NA	40	64	NA	NA	213
Turagam 2024^21^	184 (PSM)	PSM RO	Persistent AF undergoing first-time PFA, LAPWI+PVI vs PVI only; additional ablation was present in both	NA	100	64.0 vs 64.7	45 vs 44	60 vs 60	365

AF = atrial fibrillation; FU = follow-up; LA = left atrial; LAPWI = left atrial posterior wall isolation; LVEF = left ventricular ejection fraction; NA = not available; PO = prospective observational; PVI = pulmonary vein isolation; PSM = propensity score matching; PWI = posterior wall isolation; RO = retrospective observational; SA = single arm.

**Table 2:** Procedural characteristics and outcome of the included studies

Study	Success rate (%)	Mean /median applications for LAPWI	LAPW reconnection (%)	Esophageal complications (%)	ATa recurrence	Procedure (min)	Fluoroscopy time (min)
Badertscher 2024^22^	100	19	NA	NA	15/100	66 (59–77)	11 (8–14)
Davong 2023^23^	100	17.5	1/4 (redo)	0	9/45	84.1 ± 20	23.6 ± 6.8
Gunawardene 2023^17^	100	19	0 (acute)	0	13/58	91 ± 30	9 ± 5
Kueffer 2024^18^	100	20	4/26 (redo)	0	78/215	104 (79–128)	24 (17–30)
Schiavone 2024^19^	100	16	NA	0	17/142	70 (58–88)	17 (13–24)
Sohns 2023^20^	100	NA	0 (acute)	NA	1/10	62 ± 7	NA
Turagam 2024^21^	NA	NA	NA	0	26/92	78 (60–113.7)	12.7 (7.4–18)

Values are n, n/n, median (range), or mean ± SD, unless otherwise indicated.

ATa = atrial tachyarrhythmia; LAPW = left atrial posterior wall; LAPWI = left atrial posterior wall isolation; NA = not available.

### Feasibility

The success rate of LAPWI using the PFA pentaspline catheter exclusively was 100%. Two studies reported first-pass isolation of 95% and 88.8%, respectively.^19^^,^^22^ The mean/median of added PFA applications were 16 to 20. There was no reported acute LA posterior wall reconnection. Davong and colleagues^23^ reported that additional mitral isthmus ablation can be achieved in 100% of the patients using a PFA pentaspline catheter exclusively.

### AF recurrence

**Figure 2:**
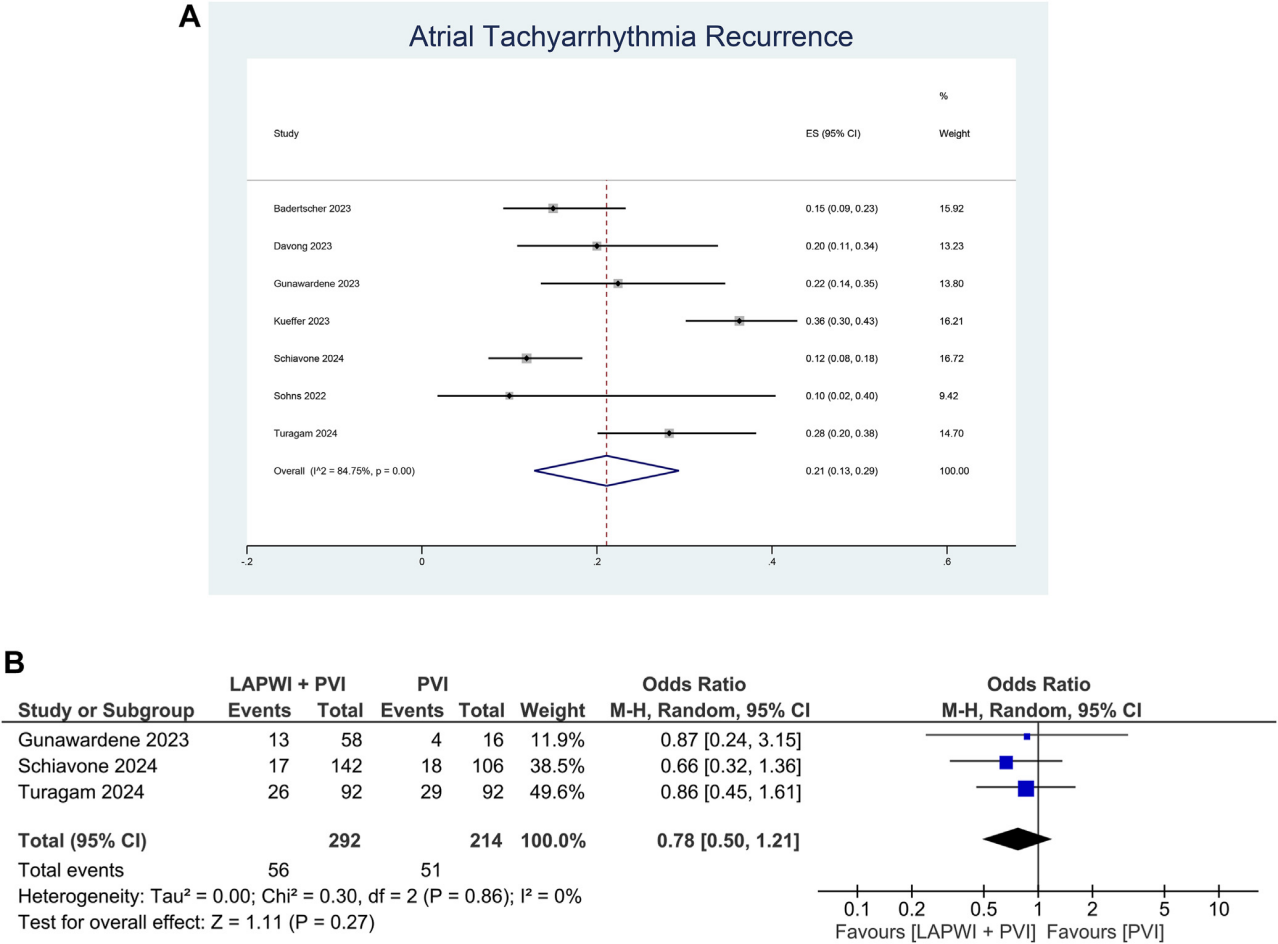
Atrial tachyarrhythmia recurrence. (A) Left atrial posterior wall isolation (LAPWI) + pulmonary vein isolation (PVI) group, (B) comparison between LAPWI+PVI group vs without LAPWI group. CI = confidence interval; ES = effect size; M-H = Mantel-Haenszel.

Meta-analysis showed that ATa recurrence was 21% (95% confidence interval [CI] 13% to 29%; I^2^ = 84.8%) in the LAPWI+PVI group (Figure 2A). Meta-regression analysis showed that age, left ventricular ejection fraction, repeat procedure, and follow-up duration did not significantly influence ATa recurrence (*P* > .05). Each 1-mm increase in LA diameter increases the chance of ATa recurrence by 6% (R^2^ = 100%, *P <* .001, I^2^ = 0%). A meta-analysis of proportion was not performed for the PVI group because of only 3 studies. The meta-analysis showed no difference in terms of ATa recurrence among LAPWI+PVI patients compared with those without LAPWI (odds ratio 0.78; 95% CI 0.50 to 1.21, *P =* .27; I^2^ = 0%, *P =* .86) (Figure 2B).

### Procedure and fluoroscopy time

**Figure 3:**
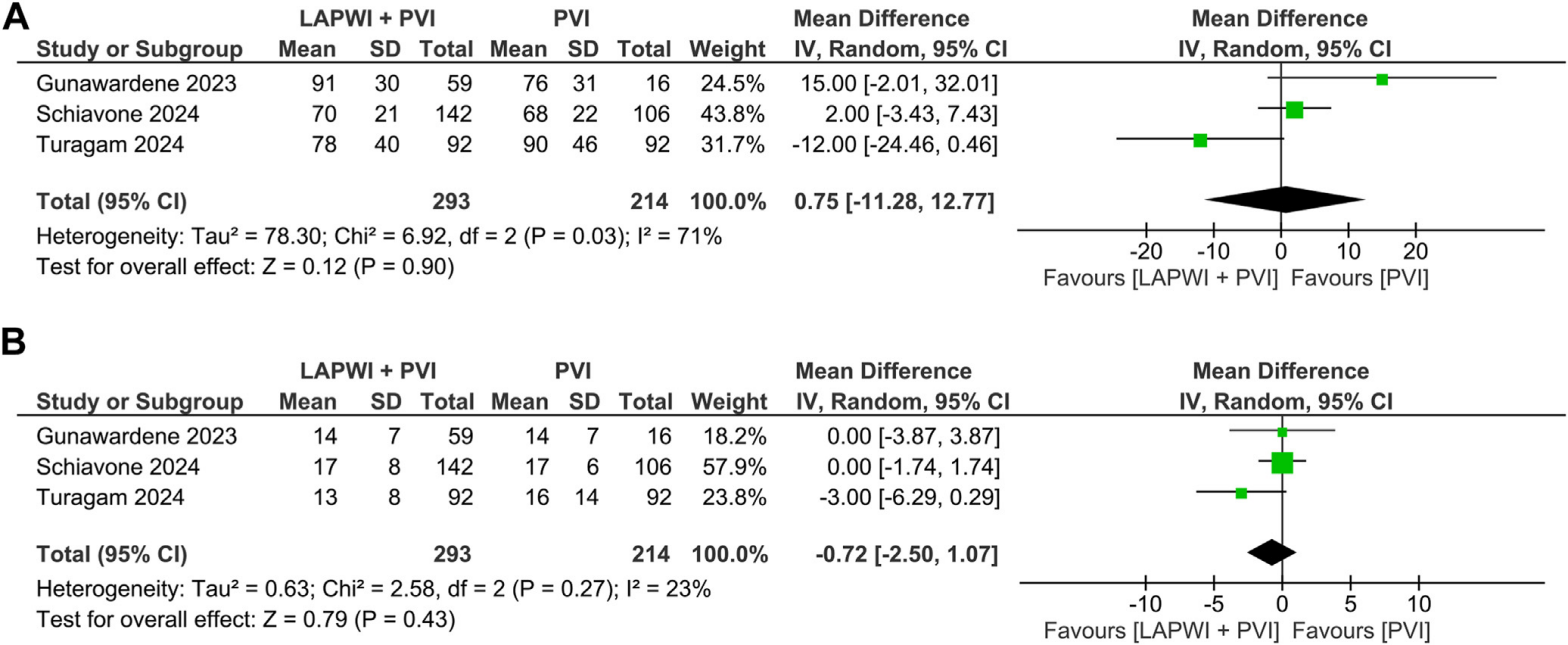
Procedure and fluoroscopy duration. (A) Procedure duration, (B) fluoroscopy duration. CI = confidence interval; IV = inverse variance; LAPWI = left atrial posterior wall isolation; PVI = pulmonary vein isolation.

Procedure time (0.75 minutes, 95% CI –11.28 to 12.77 minutes, *P =* .90; I^2^ = 71%, *P =* .03) (Figure 3A) and fluoroscopy time (0.72 minutes, 95% CI –2.50 to 1.07 minutes, *P =* .43; I^2^ = 23%, *P =* .27) (Figure 3B) were not significantly different.

## Discussion

This meta-analysis showed that LAPWI using a pentaspline catheter exclusively without additional point-by-point touch-up ablation during PFA was feasible with a 100% success rate. However, the addition of LAPWI to PVI during PFA did not reduce ATa recurrence. The recurrence of AF in LAPWI+PVI was significantly affected by LA diameter at baseline. Performing LAPWI on top of PVI does not significantly prolong the procedure or fluoroscopy time. There have been no reports of esophageal complications. Thus, LAPWI may be considered during PFA ablation without significantly prolonging the procedure, although the benefit is uncertain.

Although PVI in paroxysmal AF results in satisfactory freedom from arrhythmia, persistent AF has a high rate of recurrence.^24^ Thus, an additional strategy beyond PVI might be required, and one of popular approach is LAPWI. The CAPLA (Catheter Ablation for Persistent Atrial Fibrillation: A Multicenter Randomized Trial of Pulmonary Vein Isolation vs PVI With Posterior Left Atrial Wall Isolation) randomized controlled trial showed that LAPWI using radiofrequency energy in patients with persistent AF undergoing first-time ablation did not reduce the recurrence of atrial arrhythmia postablation compared with PVI alone while prolonging procedural time (142 minutes vs 121 minutes, *P <* .001).^25^ However, it remains uncertain whether LAPWI in repeat ablation may provide added benefits because the CAPLA trial only addresses patients with persistent AF undergoing first-time ablation, and a meta-analysis of 10 studies showed the benefit of LAPWI.^25^^,^^26^

The posterior wall of the LA lies close to esophagus; thus, performing LAPWI using radiofrequency energy prolonged the procedure time and increased the risk of esophageal complications, including fatal atrioesophageal fistula, while not providing additional benefit. PFA has myocardial tissue selectivity, thus sparing nerves, vasculature, and esophageal tissue and thus potentially avoiding esophageal complications during LAPWI.^8^, ^9^, ^10^, ^11^, ^12^, ^13^, ^14^ Additionally, PFA was shown to result in less fibrotic changes with recovered maximum strain on PV antra, LA expansion index, and LA active emptying fraction in the chronic stage after ablation compared with thermal ablation, thus preserving tissue compliance, LA reservoir, and booster pump.^9^ LAPWI creates extensive lesions on the LA and may impair its function; however, LAPWI PFA appears to prevent LA restrictive physiology.^15^ Tohoku and colleagues^7^ showed that in 360 patients undergoing PFA, 25 underwent repeat ablation, of whom 16 had atrial tachycardia. The mechanism of atrial tachycardia was macro–re-entry in all but 1 patient; 8 of 13 patients had the critical isthmus of the macro–re-entry located at the posterior wall and 4 of 13 patients had the critical isthmus located at the anterior wall. Tohoku and colleagues observed that atrial tachycardia was unexpectedly high compared with ablation using other modalities, and the substrate appeared to be associated with postablation macro–re-entry circuits. Studies that performed LAPWI+PVI during the index procedure showed that the LA posterior wall remained isolated in 75% and 85% of repeat ablations, thus effectively reducing the number of substrates at posterior wall.^18^^,^^23^ These findings suggest a potential role for prophylactic LAPWI in PFA, although our meta-analysis showed no significant difference in terms of ATa recurrence.

LAPWI using a pentaspline PFA catheter can be achieved by retracting the catheter to the flower configuration and providing overlapping applications to cover the entire posterior wall. Our study found that the mean/median of PFA applications excluding PVI ranged from 16 to 20 applications.^18^ This meta-analysis showed that LAPWI using a pentaspline PFA catheter can be achieved in 100% of cases by adding a few simple steps to the catheter maneuver that do not significantly prolong the procedure or fluoroscopy time and do not result in esophageal-related complications that are of concern in radiofrequency ablation. Badertscher and colleagues^27^ showed that the use of 3-dimensional electroanatomic mapping during PFA PVI do not significantly reduce the ATa recurrence on follow-up, while halving the procedure and LA dwell time compared with nonmapping PFA PVI. Studies have shown that LAPWI+PVI using PFA can be performed by without 3-dimensional electroanatomic mapping.^19^^,^^21^ Thus, LAPWI during PFA will not be limited to operators that prefer to use a mapping strategy. LAPWI using a pentaspline PFA catheter also did not result in added expense, because no additional equipment was required. Davong and colleagues^23^ demonstrated that mitral isthmus ablation using PFA can also be performed using a pentaspline catheter exclusively, thus possibly expanding the role of PFA in additional ablation.

We observed high heterogeneity (I^2^ = 84.8%) in the proportion of ATa recurrence in the LAPWI group. Meta-regression showed that LA diameter was significantly associated with ATa recurrence, with each 1-mm increment increasing the chance of ATa recurrence by 6%. LA size was associated with larger low-voltage areas, which predict ATa recurrence; thus, it is possible that additional ablation substrate–based ablation beyond PVI and LAPWI is more likely required as the LA diameter increases.^28^^,^^29^ There was no statistically significant difference in terms of LA diameter between the LAPWI group in Turagam and colleagues (propensity-matched cohort)^21^ and Gunawardene and colleagues,^17^ thus not affecting the comparison of primary outcome. Variables such as age, left ventricular ejection fraction, repeat procedure, and follow-up duration did not significantly influence ATa recurrence. Nevertheless, the number of studies was <10 and may not be sensitive enough to detect meaningful differences. There were also studies that also included paroxysmal AF, which was expected to have a lower recurrence rate. However, Kueffer and colleagues^18^ reported the highest recurrence rate (36%) in which one-third of the patients had paroxysmal AF. The rate of ATa recurrence in LAPWI+PVI compared with PVI were statistically not significant, with a heterogeneity of 0% based on 3 studies. While this meta-analysis showed no benefit of additional LAPWI in PFA ablation, the wide CI may indicate that additional studies are required to obtain a strong basis for recommendations.

Hemolysis during PFA ablation is a concern, likely due to the high-voltage currents affecting cell membranes. Theoretically, an increased number of PFA applications correlates with higher rates of hemolysis and hemoglobinuria and a greater risk of acute kidney injuries.^30^, ^31^, ^32^ Studies have demonstrated that more PFA applications were associated with an increased risk of periprocedural hemolysis; however, no clinically significant anemia was reported, and the incidence of acute kidney injury remained low.^30^, ^31^, ^32^, ^33^, ^34^ More than 70 PFA applications were linked to a higher risk of hemolysis, but significant kidney injury was uncommon in these patients.^30^^,^^34^ Mohanty and colleagues^32^ showed that postablation hydration (≥2 L of 0.9% sodium chloride) significantly reduces the risk of acute kidney injury in PFA. These studies suggest that an increased number of PFA applications raises the risk of hemolysis, making additional PFA applications during LAPWI a potential concern. Unfortunately, the studies included in this meta-analysis did not report hemolysis or acute kidney injury as outcomes, so the incidence of clinically significant hemolysis or acute kidney injury in patients receiving additional LAPWI remains uncertain. However, based on available studies, the incidence of clinically significant anemia or kidney injury appears limited, and postablation hydration may reduce the incidence of acute kidney injury.

### Limitations

Limitations include the small number of studies and the lack of randomized controlled trial. PFA is a relatively new technology, and exploration on additional ablation using pentaspline catheter in terms of outcome was started recently. This meta-analysis showed that LAPWI is feasible but has evidence gaps that can be filled with further randomized controlled trials comparing additional ablations, including LAPWI, with PVI alone in PFA ablation. The small number of studies also limits the power of meta-regression in detecting associations between variables, and while this may not change the association between LA diameter and ATa recurrence, additional studies may alter the meta-regression results for other variables.

### Conclusion

LAPWI using a pentaspline catheter during PFA for AF was feasible and did not prolong the procedure or fluoroscopy time; however, it did not reduce ATa recurrence. LAPWI may be considered during PFA ablation, although the benefit is uncertain.

## Disclosures

The authors have no conflicts to disclose.
